# Connected Vehicle as a Mobile Sensor for Real Time Queue Length at Signalized Intersections

**DOI:** 10.3390/s19092059

**Published:** 2019-05-02

**Authors:** Kai Gao, Farong Han, Pingping Dong, Naixue Xiong, Ronghua Du

**Affiliations:** 1 College of Automotive and Mechanical Engineering, Changsha University of Science & Technology, Changsha 410114, China; kai_g@csust.edu.cn (K.G.); hanfarong@stu.csust.edu.cn (F.H.); csdrh@csust.edu.cn (R.D.); 2 Hunan Key Laboratory of Smart Roadway and Cooperative Vehicle-Infrastructure Systems, Changsha 410114, China; 3 College of Information Science and Engineering, Hunan Normal University, Changsha 410081, China; 4 College of Intelligence and Computing, Tianjin University, Tianjin 300350, China

**Keywords:** connected vehicle, queue length, shockwave, BP neural network, penetration rate

## Abstract

With the development of intelligent transportation system (ITS) and vehicle to X (V2X), the connected vehicle is capable of sensing a great deal of useful traffic information, such as queue length at intersections. Aiming to solve the problem of existing models’ complexity and information redundancy, this paper proposes a queue length sensing model based on V2X technology, which consists of two sub-models based on shockwave sensing and back propagation (BP) neural network sensing. First, the model obtains state information of the connected vehicles and analyzes the formation process of the queue, and then it calculates the velocity of the shockwave to predict the queue length of the subsequent unconnected vehicles. Then, the neural network is trained with historical connected vehicle data, and a sub-model based on the BP neural network is established to predict the real-time queue length. Finally, the final queue length at the intersection is determined by combining the sub-models by variable weight. Simulation results show that the sensing accuracy of the combined model is proportional to the penetration rate of connected vehicles, and sensing of queue length can be achieved even in low penetration rate environments. In mixed traffic environments of connected vehicles and unconnected vehicles, the queuing length sensing model proposed in this paper has higher performance than the probability distribution (PD) model when the penetration rate is low, and it has an almost equivalent performance with higher penetration rate while the penetration rate is not needed. The proposed sensing model is more applicable for mixed traffic scenarios with much looser conditions.

## 1. Introduction

With the increasing traffic congestion problem, the role of adaptive traffic control systems (ATCS) has become more and more important. The first prerequisite for a reasonable adaptive control system is real-time—that is, the signal light can quickly and accurately reflect actual traffic conditions at the intersection and give the phase timing scheme in time [1]. The length of the entrance lane is an indispensable parameter for evaluating the traffic efficiency of an intersection, and it is also an important basis for real-time optimization of signal timing [2]. Therefore, accurate evaluation of queue length is of great significance to reducing the delay of vehicles at the intersection.

Loop and video detectors are currently the most common methods of queue length evaluation. However, the loop detector can only detect the vehicle passing information at a fixed position and cannot directly obtain the vehicle’s status dates (such as position, speed, acceleration, etc.). Video detectors can collect macro information such as traffic flow, space occupancy, and queue length [3]. The ATCS has very high requirements for the detector, and its reliability has a great impact on the control system. Once the detector fails, the control effect will plummet, and it has disadvantages of high installation and maintenance costs as well as low service life.

In order to solve the above problems, the connected vehicle as an optional "mobile detector" has been utilized [4,5,6]. The connected vehicle equipped with Global Positioning System (GPS) and wireless communication equipment can obtain the position, the speed, and other information of the vehicle, which can be used to evaluate driving time, speed, queue length, and to provide other services in traffic [7]. The connected vehicles can use vehicle to X (V2X) technology to achieve fast, accurate, and efficient transmission of information between vehicles and vehicles (V2V), vehicles and infrastructure (V2I), and vehicles and road users (V2P). At the same time, in the V2X environment, the connected vehicles or roadside units (RSU) can obtain other vehicles’ locations and speed information in real-time and among wider areas. Moreover, since the on-board device is based on the on-board individual, individual faults will not have a great impact on the whole [8,9,10,11,12]. The signal controller at the intersection can use V2I technology to obtain queue information of the entrance lanes and adjust green time in real-time. Moreover, the V2V communication and intelligent control technology can assure the vehicles cross the intersection safely without traffic signals [13]. In smart cities, a vehicle’s location prediction based on vehicle communication is utilized to get more timely and appropriate services [14]. The connectivity and performance of vehicular networks is studied in [15], which makes the V2X more available. The intrusion detection of V2X is also investigated to assure the security of V2X and traffic, including network perspective [16,17] and sensor perspective [18]. 

Despite these preparations done by the aforementioned works, the popularization of connected vehicles has been a gradual process. Although the penetration rate of connected vehicles will increase in the future, the proportion is still very small in the context of huge vehicle ownership. Therefore, how to use the information provided by a small number of connected vehicles to accurately predict the queue length becomes the key to adaptive signal timing optimization. In view of the non-linearity, the complexity, and the uncertainty of urban traffic systems, it is difficult to obtain accurate sensing results in the time period with large flow fluctuations relying on only one sensing model. The artificial neural network is an information processing technology based on the structure and the function of the brain’s neural network. It is composed of a large number of simple components connected to each other. It has a high degree of non-linearity and can perform complex logic operations [19]. By combining V2X technology and the neural network, the sensing model of queue length is established, which will further improve the sensing accuracy and provide convenience for signal control at intersections. With the development of fog computing, cloud computing, and Internet of Vehicle (IoV), machine learning algorithms can be implemented in the fog and the cloud server [20]. A data-driven intelligent framework for IoV is proposed in [21], and a game-based framework for service provision in a vehicular cloud for various types of users is studied in [22].

Based on the above advantages of V2X technology and the artificial neural network, this paper proposes a novel queue length sensing model, which includes two sub-models—the sensing model, based on shockwave and the sensing model based on the back propagation (BP) neural network. The first model uses real-time queue information of connected vehicles to determine the speed of shockwave and predict the length of subsequent unconnected vehicles. In order to consider the influence of vehicle arrival rate change on sensing results, the correction coefficient of queue length is proposed, which is determined by the flow of upstream intersection exits. The second model analyzes the historical queue length and connected vehicles distribution information and uses the nonlinear mapping characteristics of the BP neural network to establish a functional model. In view of the uncertainty of advantages and disadvantages of the two sensing models, the sensing results of the two models are weighted to obtain the predicted final queue length. VISSIM and MATLAB are used to verify the model and analyze the influence of different penetration rates on the sensing results. Absolute error and relative error are used to test the reliability of the model.

This paper is organized as follows. In Section 2, a short literature review on queue length estimation based on connected vehicles data is presented. Section 3 introduces the combined model including the shockwave-based and the BP neural network-based models. The simulation and result analysis are reported in Section 4. Finally, Section 5 presents the conclusions of this paper and suggestions of future work on this particular topic.

## 2. Literature Review

With the development of V2X technology, the connected vehicle as a "mobile sensor" to collect traffic information has become the key to traffic management. More and more people are devoted to researching the processing and the application of connected vehicle data, looking for a method that can accurately reflect the real-time state of traffic with little connected vehicle data. For example, Zheng et al. [23] use GPS trajectory data of a small number of connected vehicles or other navigation devices to predict the traffic volume at the intersection, and the model regarded vehicle arrival as the Poisson distribution to establish the maximum likelihood problem solving flow evaluation. Wang et al. [24] propose a new topological framework based on connected vehicle data, which is used to model road networks and present the propagation patterns of traffic flow; the study designs a graph recurrent neural network as an online predictor to learn the propagation patterns in the road networks. The diversification of traffic information makes the application of connected vehicle data more and more extensive.

Large amounts of connected vehicle data are collected, processed, and analyzed for traffic state evaluation [25]. Firstly, accurate traffic flow prediction in an intelligent transport system using connected vehicles is significant. EI-Sayed et al. [26] propose a new supervised learning model to capture knowledge on all possible traffic patterns. This model is a refinement of the support vector machine (SVM) kernels with a radial basis function. Goudarzi et al. [27] present a novel approach by using traffic data in a self-organizing vehicular network. The results show that the proposed model achieves high performance accuracy for predicting traffic flow. Secondly, vehicle speed prediction is important information for many applications, including electric vehicles’ power management and emission or speed limit regulation [28]. In [29], the real-time traffic information of adjacent roads is accessible and utilized for vehicle speed prediction. Thirdly, density prediction is also an important work in real-time road network state evaluation. Several other traffic measures, such as traffic speed or volume, can act as surrogate indicators for a congested situation, but density has been identified as the most important parameter to identify traffic congestion [30]. Various infrastructure-based mechanisms are proposed in [31] to estimate traffic density relying on vehicle detection devices. Furthermore, C.B. et al. [32] discuss the evaluation of a traffic congestion detection system that can detect traffic congestion in a precise way by means of a series of algorithms that reduces localized vehicular emission by rerouting vehicles. The analysis based on connected vehicle data will become the most important means of urban traffic state evaluation.

Queue length sensing based on connected vehicles is one of the applications. Vehicle arrival is often seen as a probability problem. In [33,34,35], mathematical statistics are used to calculate conditional probability distribution of queue length and the expected queue length, taking the position of the last connected vehicle in the motorcade, the queue time, and the total number of connected vehicles as inputs. Meanwhile, the calculation methods of penetration rate and arrival rate are given. The relationship between sensing error and penetration rate is analyzed in [36], and the calculation formula of sensing error is proposed. The sensing model requires that the probability functions of penetration rate and queue length of the motorcade should be determined in advance and are only suitable for unsaturated isolated intersections. The theory of shockwave describes the formation and dissipation process of queue by analyzing the changes of traffic flow density [37], which have a critical state in time and space. An and Ban et al. [38,39,40,41] determine the maximum queue length of the entrance lane based on the critical points of the changes in density and travel time. Feng et al. [42] propose an estimation of location and speed (EVLS) algorithm to determine the speed and the position of unconnected vehicles on the road by using the status information of connected vehicles. The sensing model divides the intersection entrance road into three areas: queuing area, deceleration area, and free driving area. Although the influence of arrival rate change is taken into account in this method, no specific method for determining correction parameters is given. The model is verified with a high penetration rate, which needs to be further verified if it can be applied to the transition phase of the vehicle infrastructure cooperative environment. 

Based on V2X technology, trajectory information of connected vehicles is not difficult to obtain. In [43,44], the trajectory reconstruction model is established based on location and time information of connected vehicles, and queue length evaluation and trajectory optimization are completed. However, when the penetration rate is low, the sensing results produce large errors. Similar to the idea of mathematical statistics in [33], Xu et al. [9] convert the sensing problem of queue length into the estimation problem of the number of vehicles and propose an improved interpolation algorithm to analyze the sensing results at different penetration rates. This method has a large sensing error when penetration rate is low. It assumes that the vehicle arrives with Poisson distribution but does not consider the impact of uneven distribution on the sensing results. As mentioned in [45], an estimation algorithm based on convex optimization extends the widely used linear back of queue (BoQ) curve to segment linear BoQ curve and uses the convex optimization model to estimate the segment curve in order to consider more practical situations. The effects of low penetration rate, low sampling rate, and traffic disturbance on the model are considered, and the vehicle departure information from an upstream intersection is used to improve evaluation accuracy. However, the model is too complicated and requires high data processing capability for the roadside unit.

In addition, it is more difficult to predict the queue length by relying only on the information of intelligent vehicles. Therefore, many scholars use multi-source information fusion technology to improve the accuracy of sensing. In [46,47], the data fusion of loop detectors and connected vehicles is used to build the sensing model. In [48], the data fusion of upstream and downstream detectors is used to build the discriminant models based on time occupancy rates and impulse memories. In [49], the data collected by the distributed video network are integrated to monitor and track the changes of shockwaves in real-time. Data fusion greatly improves the accuracy of sensing but also increases the complexity and the economic cost of implementation. Therefore, under the premise of low cost and low penetration rate, the evaluation model that can guarantee high sensing accuracy will have larger application scenarios. In [50] and [51], a calculation method of the minimum penetration rate is proposed, and experiments confirm that the minimum penetration rate meeting the accuracy requirement is 1%. If the expected model is more in line with the actual situation, more factors need to be considered, such as vehicle type, lane transformation, etc. This paper does not involve the influence of such factors in the results.

The contributions of the proposed method are mainly as follows: (1) a new shockwave sensing model is defined, which takes into account the influence of upstream intersection flow change on downstream queue length and gives specific correction parameters and calculation formulas; (2) the sensing method in this paper does not need to determine penetration rate in advance, which makes up for the assumption in [3,24,34,35] and is applicable to different penetration rate environments; (3) the sub-models in this paper have different application conditions. According to the comprehensive analysis of the proposed weight calculation method, the combined model is more robust than a single model. The above characteristics allow this method to have wider application scenarios and lower application costs.

## 3. Queue Length Sensing Model

### 3.1. Basic Conditions

The queue length sensing method proposed in this paper needs to meet some following basic conditions:

(1) Connected vehicles must be equipped with GPS and wireless transmission equipment;
(2) It is assumed that roadside units (such as signal controller) can accurately obtain information such as connected vehicle’s ID and location;
(3) The motorcade in each entrance lane must contain at least one connected vehicle;
(4) This paper assumes that all participating vehicles (including connected vehicles and unconnected vehicles) are standard vehicles.

The first condition requires the GPS to accurately locate the lane of a vehicle, and it requires the wireless transmission device to formulate an emergency treatment plan to ensure the timely transmission of information to the roadside unit in case of equipment failure. The second condition requires the roadside unit to receive information sent by connected vehicles in real-time and to accurately process the data effectively and feed back to vehicles in time. The third condition requires each motorcade to include at least one connected vehicle, because the method studied in this paper is applicable to the vehicle infrastructure cooperative environment. This paper does not consider the queue sensing of all unconnected vehicles. Some literature refers to the use of traditional detection methods (such as loop detector) to deal with this situation and the use of information fusion technology to achieve the sensing work. The fourth condition assumes that all vehicles are standard vehicles, which means a vehicle’s type is not considered. However, in the actual situation, different vehicle types need to be converted according to the vehicle conversion coefficient. The above basic conditions can reduce difficulty and complexity of problem research to a large extent. In the future, we hope to reduce the limitation of hypothesis conditions and expand this method to a larger scope of application.

The four basic conditions above imply that the queue length sensing model has some limitations. Firstly, the sensing scene must be the signalized intersections. Secondly, the model is only applicable to mixed traffic environments, where both connected and unconnected vehicles exist in the motorcade, or traffic environments where all connected vehicles exist. Finally, because the model assumes that vehicles pass through the intersection in the form of waves, the model is suitable for predicting the length of the queue formed in the entrance lane.

**Figure 1:**
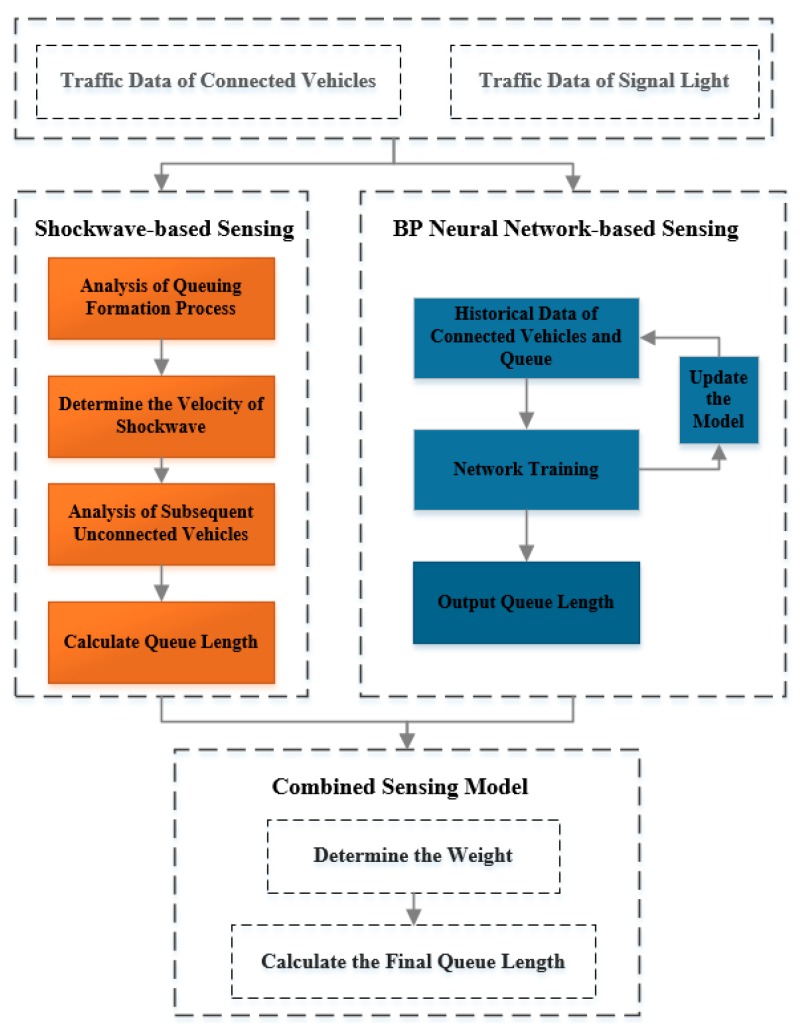
The overall structure of the sensing model.

Based on the above preconditions and limitations, this paper proposes a real-time sensing model for queue length. The overall structure of this model is shown in Figure 1. The shockwave-based sensing and the BP neural network-based sensing respectively estimate the queue length and combine the advantages of two sub-models in different penetration rates to construct the combined sensing model, which has higher accuracy.

### 3.2. Sensing Model Based on Shockwave

The queue length sensing based on shockwave uses V2X technology to determine total queue length of each entrance lane during the red-light period, which is used as the basis for signal optimization. Queuing vehicles are defined as vehicles that arrive at the intersection entrance load with the speed reduced to zero and wait for the green light to pass. The connected vehicle needs to send the following information to the roadside unit: vehicle’s ID, location, queue time, and speed. Assuming that the vehicle arrives in a Poisson distribution, forms a queue with the corresponding arrival rate, and passes back in the form of a wave during the red light, the wave is defined as a shockwave. The key is to determine the velocity of the shockwave using connected vehicles’ information. Based on the number of connected vehicles in the motorcade, this paper takes the single-lane scenario as an example for simplified analysis.

Scenario 1: There is only one connected vehicle in the motorcade (n=1).

**Figure 2:**
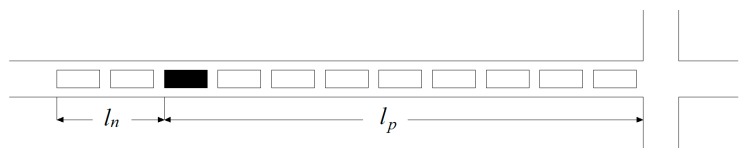
Schematic diagram of one connected vehicle.

Figure 2 shows the schematic diagram of only one connected vehicle in the motorcade; the solid rectangle is the connected vehicle p, and n represents the number of connected vehicle. Penetration rate is defined as the ratio of the number of connected vehicles in a motorcade to the total number of vehicles. The predicted time period is the red light period of each signal cycle, i.e., ($t_{r}$, $t_{f}$). $t_{r}$ represents the start time of the red light, $t_{f}$ represents the end time of the red light. In Figure 2, $l_{p}$ represents the queue length in front of the connected vehicle, which is determined by the difference between the position of the connected vehicle and the stop line. $l_{n}$ represents the queue length of the unconnected vehicle behind the connected vehicle. The velocity of the shockwave can be determined by Equation (1):

(1) $$v_{1}=\frac{l_{p}}{t_{1}-t_{r}}.$$

where $t_{1}$ is the stop time of the connected vehicle. The total queue length is equal to the sum of the queue length of all vehicles in front of the connected vehicle (including the connected vehicle) and the queue length of the unconnected vehicles arriving at the rest red light time, which can be calculated by Equations (2) and (3):

(2) $$l_{n}=v_{1}\left(t_{f}-t_{1}\right).$$

(3) $$l=l_{p}+v_{1}(t_{f}-t_{1}).$$

where l represents the total queue length of the lane. Since there is only one connected vehicle in Scenario 1, it is prone to a large deviation of the sensing results.

Scenario 2: There are at least two connected vehicles in the motorcade (n≥2).

**Figure 3:**
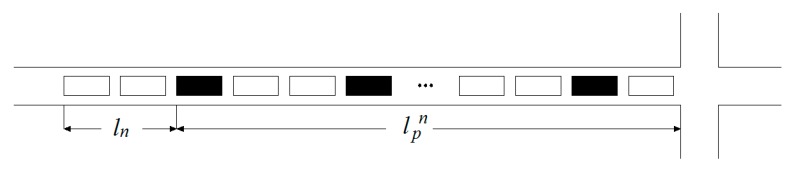
Schematic diagram of *n* connected vehicles.

There are n connected vehicles in the motorcade as shown in Figure 3 and the velocity of the shockwave is determined by Equation (4):

(4) $$v_{n}=\frac{1}{n-1}\sum_{i=1}^{n-1}\frac{l_{p}^{n}-l_{p}^{i}}{t_{n}-t_{i}}\;\;\left(i=1,2,\cdots ,n-1\right).$$

where i is the connected vehicle, $l_{p}^{n}$ is the queue length of the last connected vehicle, $l_{p}^{i}$ is the queue length before the i connected vehicle, which is determined by the difference between the position of the last connected vehicle and the stop line, $t_{n}$ is the stop time of the nth connected vehicle, and $t_{i}$ is the stop time of the i connected vehicle. The queue length of unconnected vehicles after the nth connected vehicle is determined by Equation (5):

(5) $$l_{n}=v_{n}\left(t_{f}-t_{n}\right).$$

The total queue length of the entrance lane is determined by Equation (6):

(6) $$l=l_{p}^{n}+v_{n}\left(t_{f}-t_{n}\right).$$

However, vehicle arrival is random and non-uniform in the actual traffic environment. When the last connected vehicle enters the motorcade, the subsequent unconnected vehicles may form a queue with different arrival rates. If the original arrival rate is still used to predict the queue length of subsequent vehicles, the results may be overestimated or underestimated. For example, when the arrival rate decreases, the predicted value of queue length will be larger, and vice versa. Therefore, a modified parameter *r* is proposed in this paper to modify the predicted values of Equations (3) and (6).

**Figure 4:**
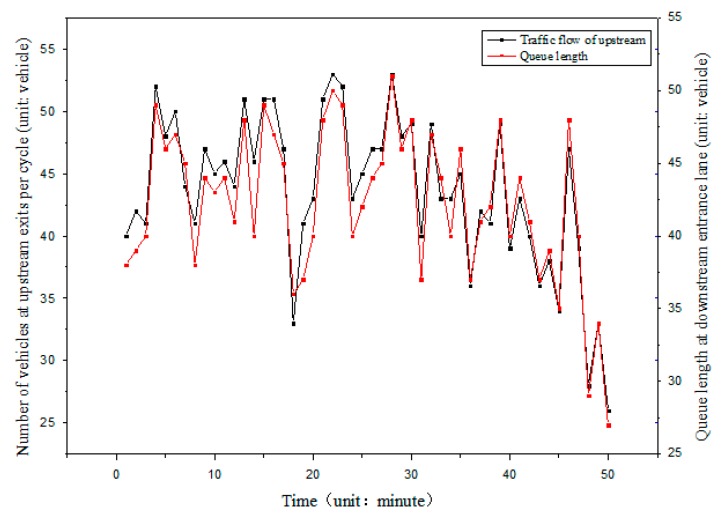
Relationship between traffic volume and queue length.

**Figure 5:**
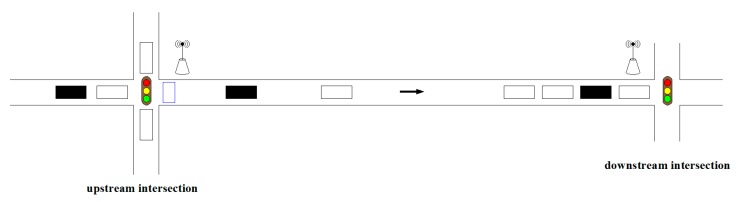
Schematic diagram of upstream and downstream traffic flow.

In urban roads, the distance between upstream and downstream intersections is relatively close, and the arrival rate of vehicles at the downstream intersection has a great relationship with traffic flow of an upstream intersection. As shown in Figure 4, the queue length of a downstream lane changes with upstream traffic flow. The predictive value of queue length can be corrected by using the connected vehicle’s information received by the roadside units at the upstream intersection. The schematic diagram of upstream and downstream traffic flow is shown in Figure 5.

In Figure 5, the blue rectangle represents the loop detector, which is arranged in the exit lane at the intersection. It is worth noting that the loop does not need to be reinstalled in practical applications—the existing loop detector, such as the loop used to detect whether a vehicle is running a red light, can be used. When the connected vehicle passes through the loop detector, the roadside unit will receive the ID and the time (the time passing through the loop) information sent by this vehicle and will record the number of unconnected vehicles between two connected vehicles. The formula for calculating correction parameter *r* is as shown in Equation (7):

**Remark 1:**  Since the actual traffic arrival is stochastic and generally nonhomogeneous, it is possible that the last connected vehicles join the queue in a relatively short time, but the arrival rate of unconnected vehicles drops significantly afterwards. In this case, if the velocity of the shockwave is multiplied by the difference between the arrival time of the last connected vehicle and the end time of the red light, the queue length can be overestimated. Therefore, it is significant to consider the correction parameter r.

(7) $$r=\frac{\frac{q_{k,k+1}}{t_{k+1}-t_{k}}}{\frac{q_{k-1,k}}{t_{k}-t_{k-1}}}.$$

where *k* is the connected vehicle passing the loop detector, $q_{k,k+1}$ is the number of unconnected vehicles between *k* and *k* + 1 connected vehicles, and $t_{k}$ is the time when the *k* connected vehicle passes through the loop detector. It can be seen from Equation (7) that, when the upstream flow increases, the rate *r* > 1, and the downstream arrival rate increases. When the upstream flow decreases, *r* < 1, and the downstream arrival rate decreases. Therefore, the modified formula for calculating the total queue length is shown in Equation (8):

(8) $$l_{1}=\left\{ \begin{array}{l} l_{p}+v_{1}r\left(t_{f}-t_{1}\right),\;\;\left(n=1\right);\\ l_{p}^{n}+v_{n}r\left(t_{f}-t_{n}\right),\;\;\left(n\geq 2\right). \end{array}\right.$$

In the simulation and in actual operation, this parameter needs to be properly adjusted by considering the distance and the average speed of upstream and downstream intersections.

### 3.3. Sensing Model Based on BP Neural Network

The queue length of the intersection is affected by many factors and has great randomness. A neural network is composed of many simple information processing elements of neurons or nodes, which can automatically adjust the connection weight between internal neurons to match the input–output response relationship and has the advantage of nonlinear mapping.

**Figure 6:**
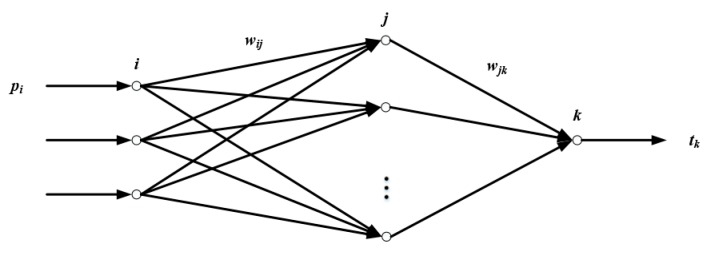
Simplified back propagation (BP) neural network diagram.

The BP neural network is composed of two parts, the forward transmission of information and the back propagation of error. Figure 6 is a simplified BP neural network diagram. In the figure, *i* is the input layer neuron, with a total of *r*; *j* is the hidden layer neuron, with a total number of s1; *k* is the output layer neuron, with a total number of s2; $p_{i}$ is the sample input, $t_{k}$ is the sample output, $w_{ij}$ is the weight between input layer and hidden layer, and $w_{jk}$ is the weight between hidden layer and output layer. The specific process of the BP neural network is as follows.

Step 1: Forward transmission of information. It is known that the weight between neurons is *w*, the deviation is *b*, and the neuron output value is *a*. Then, the output of the *j* neuron in the hidden layer is:

(9) $$a_{j}=f1(\sum_{i=1}^{r}w_{ij}p_{i}+b_{j})\;\;(j=1,2,\cdots ,s1).$$

The output of the k neuron in the output layer is:

(10) $$a_{k}=f2(\sum_{j=1}^{s1}w_{jk}a_{j}+b_{k})\;\;(k=1,2,\cdots ,s2).$$

where f1 is the activation function between input and hidden layer, and f2 is the activation function between hidden and output layer.

Step 2: Back propagation of the error. If the desired output value is not obtained at the output layer, the network calculates the error variation value of the output layer and propagates back. The error is transmitted back along the original connection through the network to adjust the weight of each layer of neurons until the desired value is reached. The error function is:

(11) $$E(w,b)=\frac{1}{2}\sum_{k=1}^{s2}{\left(t_{k}-a_{k}\right)}^{2}.$$

The gradient descent method is used to calculate the weight change and the back propagation of error. For the weight from j input to k output:

(12) $$\Delta w_{jk}=-\eta \frac{\partial E}{\partial w_{jk}}=\eta \cdot \delta_{jk}\cdot a_{j}.$$

where,

(13) $$\delta_{jk}=(t_{k}-a_{k})\cdot f2^{\prime }.$$

thus,

(14) $$\Delta b_{k}=-\eta \frac{\partial E}{\partial b_{k}}=\eta \cdot \delta_{jk}.$$

For the weight from i input to j output:

(15) $$\Delta w_{ij}=-\eta \frac{\partial E}{\partial w_{ij}}=\eta \cdot \delta_{ij}\cdot p_{i}.$$

where,

(16) $$\delta_{ij}=e_{j}\cdot f1^{\prime },\;e_{j}=\sum_{k=1}^{s2}\delta_{jk}w_{jk},\;\delta_{jk}=(t_{k}-a_{k})\cdot f2^{\prime }.$$

thus,

(17) $$\Delta b_{j}=\eta \delta_{ij}.$$

Thus, the queue length $l_{2}$ of the sub-model based on the BP neural network is given as Equation (18).

(18) $$l_{2}=a_{k}.$$

This paper assumes that the queue length sensing model has three inputs, $p_{1}$, $p_{2}$, and $p_{3}$. They are the distance between the last connected vehicle and the stop line, the stop time of the last connected vehicle, and the number of connected vehicles in the motorcade. The queue length is the only output $t_{1}$, and the number of neurons in the hidden layer can be determined by experiment or experience.

### 3.4. Weight Calculation and Reliability Test of Combined Model

Combining the advantages of the two sub-models, the weight of the sub-model is determined by the ratio of the stop time of the last connected vehicle to the red light time. The formula is as follows:

(19) $$L=\alpha l_{1}+(1-\alpha )l_{2}.$$

(20) $$\alpha =\frac{t-t_{r}}{t_{f}-t_{r}}.$$

where *L* is the final queue length, $l_{1}$ is the predicted value based on the shockwave, $l_{2}$ is the predicted value based on the BP neural network, α is the weight of the shockwave model, and *t* is the stop time of the last connected vehicle.

The accuracy of the combined sensing model is tested by two error indices, namely absolute error $A_{E}$ and relative error $R_{E}$. The specific calculation formula is as follows:

(21) $$A_{E}=\left|y_{j}-{\hat{y}}_{j}\right|.$$

(22) $$R_{E}=\frac{\left|y_{j}-{\hat{y}}_{j}\right|}{y_{j}}\times 100\%.$$

where $y_{j}$ represents the actual value of queue length in *j* cycle and ${\hat{y}}_{j}$ represents the predictive value of queue length in *j* cycle.

### 3.5. Analysis of Model’s Time Complexity 

Based on the description of the shockwave and the BP network combined sensing model, the queue length algorithm at signalized intersections is shown as Algorithm 1.

**Algorithm:** Queue Length Sensing Algorithm at Signalized Intersection
**Input:** $l_{1}$, the predicted queue length based on shockwave; $l_{2}$, the predictive queue length from BP neural network; α, the weight of shockwave model; $v_{n}$, velocity of shockwave; *n* is the number of connected vehicles in the queue; $l_{p}$, the queue length in front of the last connected vehicle; $t_{f}$, the end time of red light; $t_{r}$,the start time of red light; $t_{1}$, the stop time of connected vehicle; $p_{1}$, the distance between the last connected vehicle and the stop line; $p_{2}$, stop time of the last connected vehicle; $p_{3}$, the number of connected vehicles in the motorcade.
Output:1: L, queue length estimate by combined sensing model.2: **Algorithm begin:**3: Computing the queue length estimate $l_{1}$ based on shockwave,4: **if**
n==1
**then**5:  $l_{1}=l_{p}+v_{1}r(t_{f}-t_{1})$
6: where $v_{1}=\frac{l_{p}}{t_{1}-t_{r}}$;7: $r=\frac{\frac{q_{k,k+1}}{t_{k+1}-t_{k}}}{\frac{q_{k-1,k}}{t_{k}-t_{k-1}}}$
8: **else**9:   $l_{1}=l_{p}^{n}+v_{n}r(t_{f}-t_{n})$
10: where $v_{n}=\frac{1}{n-1}\sum_{i=1}^{n-1}\frac{l_{p}^{n}-l_{p}^{i}}{t_{n}-t_{i}}\;\;(i=1,2,\cdots ,n-1)$
11: **end if**12: BP neural network utilized to sensing the queue length based on learning of history records,13: $a_{k}=f2(\sum_{j=1}^{s1}w_{jk}a_{j}+b_{k})\;\;(k=1,2,\cdots ,s2)$. where $a_{j}$ is the output of hidden layer, $a_{j}=f1(\sum_{i=1}^{r}w_{ij}p_{i}+b_{j})\;\;(j=1,2,\cdots ,s1)$. f1 and f2 is activation function among the input, hidden and output layers.14: compute the combined sensing queue length,15: $L=\alpha l_{1}+(1-\alpha )l_{2}$.16: where $\alpha =\frac{t-t_{r}}{t_{f}-t_{r}}$ and $l_{2}=a_{k}$
17: return *L*18: **Algorithm end**

The information used in the sensing algorithm is given at the beginning, which can be collected by the road side unit (RSU) via V2X. The sensing algorithm is running on the central processing unit (CPU) of the RSU. To evaluate the proposed algorithm’s performance, the storage and the time complexity are analyzed. Moreover, the storage and the time complexity of a probability distribution (PD) model proposed in [3x] is utilized to compare with the algorithm proposed in this manuscript. 

The time complexity of the combined sensing model can be calculated partly according to Equation (19). The time complexity of the shockwave model is as follows:

(23) $$Ο\left(l\right)=Ο\left(l_{p}^{n}\right)+Ο\left(v_{n}\right)\times Ο\left(r\right)\times Ο\left(t_{f}-t_{n}\right)$$

where $Ο\left(l_{p}^{n}\right)=Ο\left(1\right)$, $Ο\left(v_{n}\right)=Ο\left(n\right)$, Ο(r)=Ο(2n), and $Ο\left(t_{f}-t_{n}\right)=Ο\left(1\right)$, thus the time complexity of the shockwave model is $Ο\left(n^{2}\right)$. *n* is the number of connected vehicles collected at the RSU.

Then, the time complexity of the BP sensing model is $Ο\left(l_{2}\right)=Ο\left(r\times s1\times s2\right)$, where *r* is the number of the input layer neuron, *s*1 is the number of the hidden layer neuron, and *s*2 is the number of the output layer neuron. Thus, the time complexity of the combined sensing model can be expressed by the following equation:

(24) Ο(L)=Ο(1)+Ο(n)×Ο(2n)×Ο(1)+Ο(r×s1×s2)

The second part is related to the structure of the BP network. The time complexity will not increase with the number of vehicles. Once the structure of the BP network is determined, the time complexity of this sub-model is a constant. From this point of view, the time complexity of the combined sensing model is $Ο\left(n^{2}\right)$.

**Remark 2:**  *The number of connected vehicles is not very large because the penetration rate keeps in a relatively low level for a long period. Additionally, the detection area before the stop line in the intersection is limited, and the number of vehicles is not very large since the penetration rate becomes high in the far future.*

## 4. Simulation and Result Analysis

To verify the effectiveness of the model proposed in this paper, a vehicle infrastructure cooperative environment is built in VISSIM to obtain real-time vehicle status information, signal transformation information, and upstream exit traffic volume information. MATLAB programmable environment is used to verify the two sub-models, and the influence of the model on sensing results of queue length under different penetration rates (*ρ*) of connected vehicles is analyzed. In order to better illustrate the performance of the method proposed in this paper, a comparative analysis is made between this model and the PD model [3]. The results show that the proposed sensing model is more suitable for mixed traffic environments with low penetration rates.

### 4.1. Model Validation Based on Shockwave

A road network is built in VISSIM in which the distance between upstream and downstream intersections is 500 m, and a loop detector is set at the upstream exit. The time interval for the connected vehicle to send information is 0.2 s. The simulation duration is 1 h, the cycle length is 70 s, and the vehicle data of 10 cycles are taken as the basis for verification analysis.

**Remark 3:**  *The connected vehicles broadcast information periodically, including ID, location, time, and speed, via basic safety message (BSM), which is defined in SAE J2735. In many investigations, vehicles typically broadcast BSMs at an interval of 100 to 300 ms [52,53]. Without loss of generality, the time interval for the connected vehicle to send information is set as 0.2 s (200 ms) in our simulation.*

**Figure 7:**
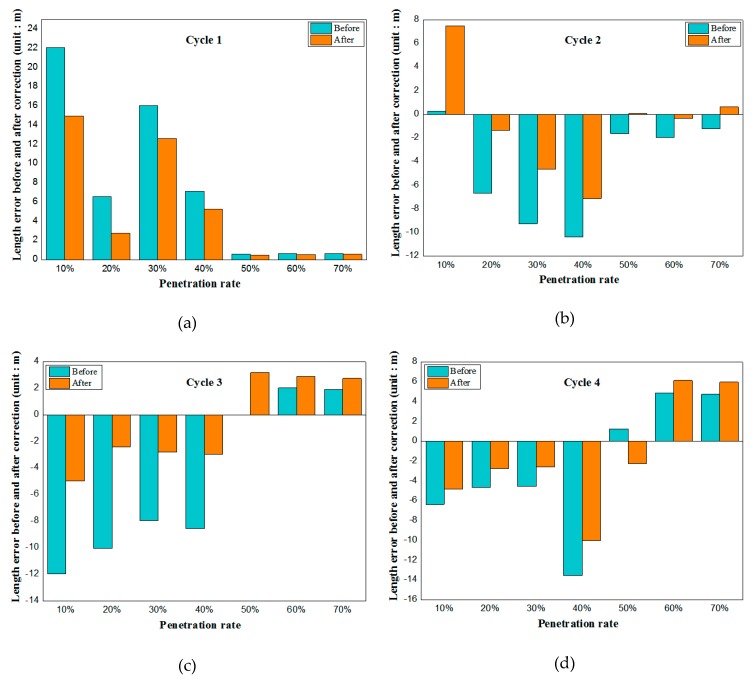
Queue length error before and after correction in cycle 1 (**a**) and queue length error before and after correction in cycle 2 (**b**) and queue length error before and after correction in cycle 3 (**c**) and queue length error before and after correction in cycle 4 (**d**).

One of the major contributions of this paper is the sensing of queue length with appreciable estimation accuracy, even when the penetration rate is low and the traffic flow changes greatly. A correction is introduced in the sensing model to remedy these cases. The effect of correction is studied in the simulation. Figure 7 shows the comparison of predicted values before and after the correction parameter. It reveals that the prediction accuracy is significantly improved after considering the modified parameters when the penetration rate is lower than 50%. However, when the penetration rate is greater than 50%, the influence of the modified parameters on the predicted value is not obvious, even worse than before the modification (see Figure 7c,d). This is because when the penetration rate is large, the position of the last connected vehicle in the motorcade is close to the actual queue length, and the change of vehicle arrival rate has little influence on the prediction result. Even when the change of arrival rate is taken into account, the queue length of unconnected vehicles arriving during the remaining red light period is estimated to be too high or too low, resulting in a larger error. Therefore, it is necessary to state here that the modified parameter *r* proposed in this paper needs to be considered when the penetration rate is less than 50% but not when the penetration rate is higher than 50%.

**Figure 8:**
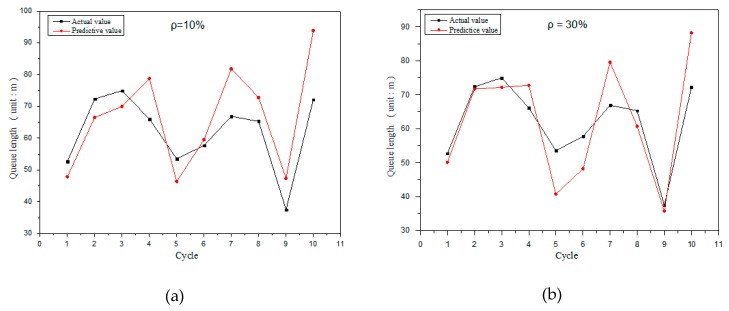

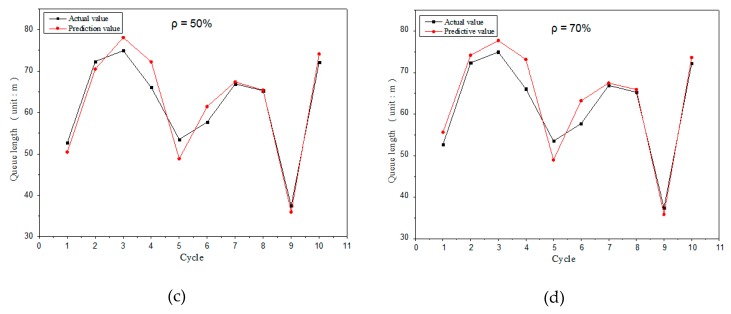
Queue length at 10% penetration rate (**a**) and queue length at 30% penetration rate (**b**) and queue length at 50% penetration rate (**c**) and queue length at 70% penetration rate (**d**).

The results of the model validation are shown in Figure 8. The sensing results of the model under different penetration rates are analyzed, and the penetration rates of Figure 8a–d are 10%, 30%, 50%, and 70%, respectively. It can be seen from the figure that the predicted queue length is obviously closer to actual queue length at a higher penetration rate, and the sensing accuracy of the model is higher.

It can be found from the analysis of Figure 8a,b that the sensing accuracy is higher in the fifth and the sixth cycle when penetration rate is lower. This is because in cycles five and six, the last connected vehicle in Figure 8a is closer to the end of the motorcade, while the last connected vehicle in Figure 8b is closer to the front of the motorcade. Therefore, the sensing model based on the shockwave mainly relies on the information provided by the last connected vehicle in the motorcade. When the penetration rate is low and the connected vehicle is located in the front of motorcade, the sensing accuracy will be reduced. Thus, this model is more suitable for the sensing of queue length when the penetration rate is high. In order to make up for the shortcomings of this model, a queue length sensing model based on a neural network is proposed to balance the sensing error caused by the shockwave model.

The simulation results in Figure 8 show that the sensing accuracy changes in different cycles when the penetration rate is determined. The reason is that the location of the last connected vehicle is different in every cycle. The location of connected vehicles is random in the simulation. Thus, the subsequent length evaluation of the unconnected vehicle is greatly affected by the arrival rate. More specifically, when the upstream traffic flow rate changes greatly, the downstream queue length estimation will be overestimated or underestimated, which may lead to bigger deviation in the actual queue length. In our sensing model, the location of connected vehicles is unknown in advance and is distributed randomly, which is close to the real traffic situation. 

### 4.2. Model Validation Based on BP Neural Network

The BP neural network is a widely used network model. In order to ensure the robustness of the model and the comprehensiveness of queuing information, one-week historical data of connected vehicles and queues are collected to build and train the model; the first 70% is used as the training data, 15% as the test data, and 15% as the verification data. In addition, the model is updated once a week to accommodate dynamically changing traffic flow. The single hidden layer network structure is adopted in the sensing model, with three nodes in the input layer and one node in the output layer. In this paper, the number of neurons in the hidden layer is set as 10 by the experimental method, which has a great impact on the network performance. If the number is too small, the accuracy of the sensing model will be reduced; if the number is too large, the training time of the network will be increased, and the sensing accuracy will be affected.

In addition, the selection of the training function and the transfer function also affects the performance. In this paper, *tansig* and *logsig* functions are selected as the transfer functions between the input layer and the hidden layer and between the hidden layer and the output layer, respectively. *Trainlm* is selected as the training function. After the network is trained, the predicted output queue length is obtained based on real-time connected vehicles data.

**Figure 9:**
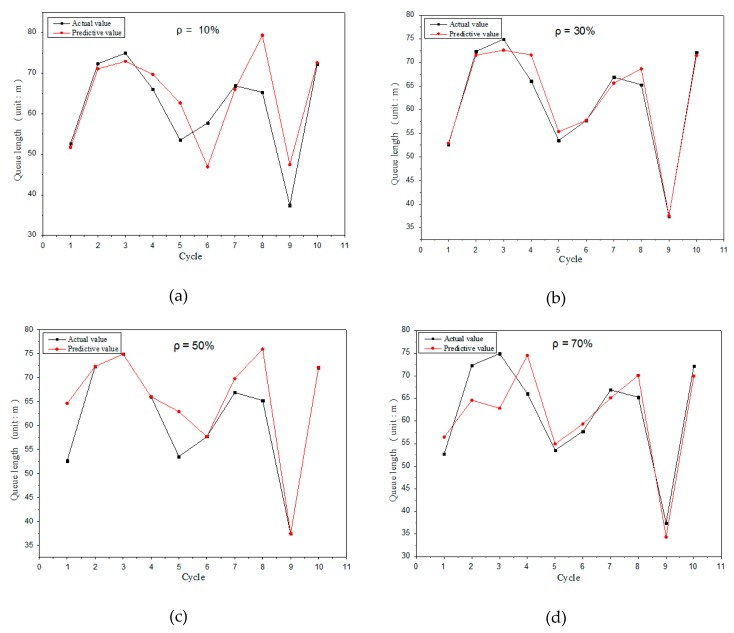
Queue length at 10% penetration rate (**a**) and queue length at 30% penetration rate (**b**) and queue length at 50% penetration rate (**c**) and queue length at 70% penetration rate (**d**).

Figure 9 shows the sensing results of the sensing model based on the BP neural network at different penetration rates. With the increase of penetration rate, the accuracy is improved. Although the sensing error of some cycles is large in Figure 9c,d, the sensing result of more cycles is almost equal to actual queue length. The sensing model based on the BP neural network is completely dependent on the information sent by connected vehicles, especially the state information of the last connected vehicle, thus the model can achieve high sensing accuracy in the traffic environments with low penetration rates.

### 4.3. Accuracy Analysis of Combined Sensing Model

The sensing model based on the shockwave is suitable for the connected vehicles when the distribution is later in the motorcade or the penetration rate is higher, because the shockwave at this time can better reflect the formation process of the queue state of the entrance lane. The BP neural network model with characteristics of nonlinear mapping can more accurately predict the queue length of mixed traffic flow when the connected vehicle is in the front of the motorcade and the penetration rate is low. In view of the uncertainty of the sensing environment, the final sensing results are obtained by weighting the sensing results of the two sub-models. The sensing results can give full play to the advantages of the two sub-models and can balance the sensing errors of the sub-models to a certain extent. The combined model is very suitable for intersections with large dynamic change of traffic flow in urban roads and can provide more convenience for traffic signal management and control.

**Figure 10:**
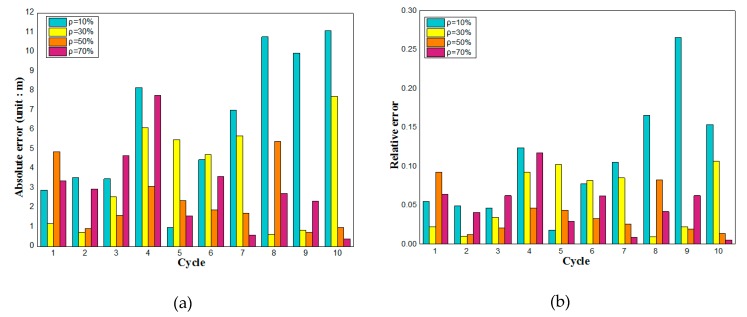
Absolute error of combined sensing model (**a**) and relative error of combined sensing model (**b**).

Figure 10 is the error analysis diagram of the combined model. Figure 10a is the absolute error, and Figure 10b is the relative error. The sensing effect of the combined model may be worse than that of one of the sub-models in the partial period, but the sensing effect of the model in the large range and in multiple stages is better than that of either of the sub-models. The sensing results under different penetration rates are analyzed experimentally. According to Figure 10, when the penetration rate increases, the sensing error significantly decreases. When the penetration rate is 70%, the predicted result is almost equal to the actual queue length. If the road driving vehicles reach a higher penetration rate, the sensing accuracy will be higher.

It can be seen from the relative error diagram that the accuracy of the combined sensing model can reach 95% at a high penetration rate, except for individual cycles. Even when the penetration rate is very low, the accuracy can reach 85%. This feature of the model is very suitable for the gradual popularization of connected vehicles. In the transition stage from unconnected vehicles to connected vehicles, this model is of great significance for signal processing at intersections.

### 4.4. Comparison and Analysis with PD Model

In order to prove the universality of the method proposed in this paper, connected vehicle data of 100 cycles are collected for analysis. At the same time, the proposed model is compared with the PD model to illustrate sensing performance. The PD model establishes the probability density function of the connected vehicle and determines the expected queue length by calculating the conditional probability distribution of the queuing vehicle. A discrete wavelet transform (DWT) is applied to enhance the proposed queue estimation to be more accurate and consistent, regardless of the randomness in the penetration rate. The comparison results are shown in the figure below.

**Figure 11:**
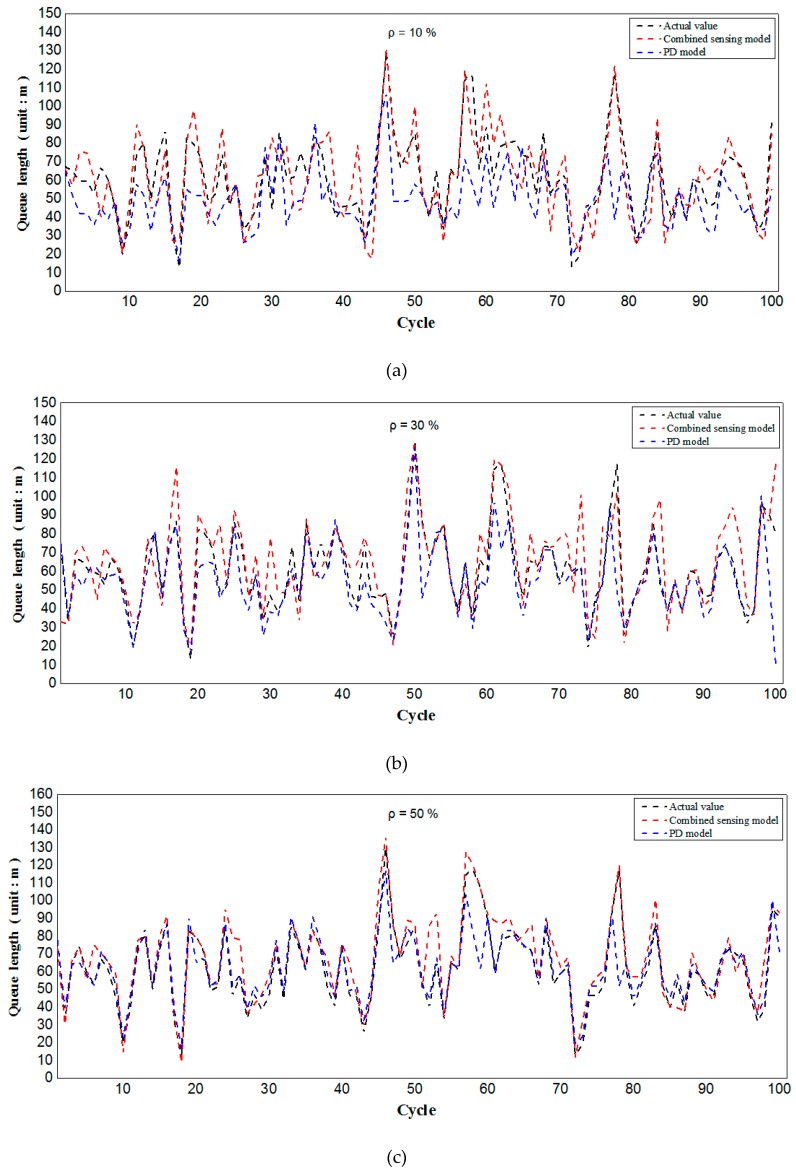

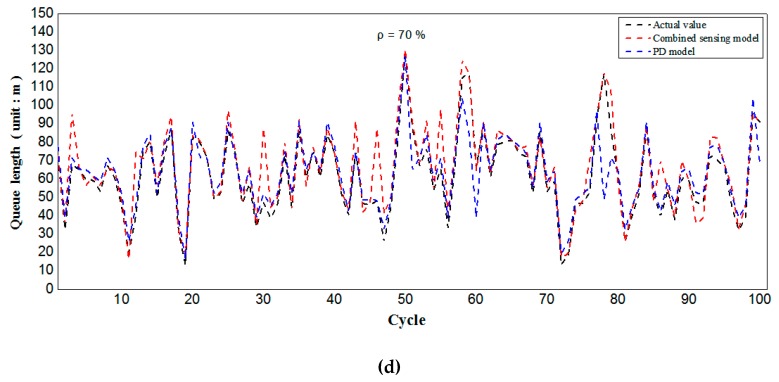
Comparison of queue length at 10% penetration rate (**a**) and comparison of queue length at 30% penetration rate (**b**) and comparison of queue length at 50% penetration rate (**c**) and comparison of queue length at 70% penetration rate (**d**).

Figure 11 analyzes the comparison results of the combined sensing model with the PD model at different penetration rates. It can be seen from Figure 11c,d that when the penetration rate is high, the predicted value of the combined model and the PD model in this paper is basically equal to the actual queue length. However, when the penetration rate is low (as shown in Figure 11a,b), both the combined model and the PD model have certain errors, but the PD model has a worse effect, which is particularly obvious in Figure 11a. On the one hand, this is because when the penetration rate is low, no moving connected vehicle is detected in the PD model, thus queue length is underestimated. On the other hand, compared with the PD model, the combined model considers the impact of upstream flow rate change on downstream queue length in low penetration rates, which is necessary for real-time length evaluation. Furthermore, it is expected that the combined sensing model in this paper can comprehensively analyze two sub-models and integrate the multi-source traffic information to achieve higher evaluation accuracy. In addition, the PD model assumes that the penetration rate of connected vehicles is known, and the prediction result relies too much on this value. If penetration rate changes greatly with time, the prediction value of this model will produce a large error.

In general, the combined model can sense the queue length with high accuracy in mixed traffic environments. Even at low penetration rates (e.g., 10%), the model can achieve a sensing accuracy of 85%. However, this model is subject to certain limitations, such as the inclusion of connected vehicles in the motorcade. When the last connected vehicle in the motorcade is in front, the sensing accuracy of this model will be affected to some extent. Even so, the sensing model in this paper is still of great application value in flow prediction and signal management based on connected vehicles.

## 5. Conclusions

In this paper, we propose a queue length sensing model suitable for mixed traffic environments. Simulation results show that the model has a high sensing accuracy in traffic environments with low penetration rates and variable traffic flow, benefiting from the correction of the shockwave and from learning history records. The simulation reveals that the sensing accuracy is proportional to the penetration rate. Compared with most existing queue estimations from connected vehicle technology for pre-timed signals, the proposed sensing model in our manuscript can be applied to adaptive signal control in the intersection, which will promote the traffic throughput and efficiency. The proposed sensing model has higher performance than the PD model when the penetration rate is low and almost equivalent performance with higher penetration rates. While the penetration rate is not needed in the proposed model, the combined sensing model is more applicable for mixed traffic scenarios (both under-saturated and saturated conditions) with much looser conditions. 

It requires that there is at least one connected vehicle in a cycle. Moreover, the estimate accuracy may be affected by the locations of the connected vehicles, especially when all of the vehicles stop in the front part of the queue. It is interesting and valuable to investigate an algorithm that is not susceptible to the location of connected vehicles even when the penetration rate is low.

## References

[1] Chandan K., Seco A.M., Silva A.B. (2017). Real-time traffic signal control for isolated intersection, using car-following logic under connected vehicle environment. Transp. Res. Procedia.

[2] Argote J., Christofa E., Xuan Y., Skabardonis A. Estimation of measures of effectiveness based on connected vehicle data. Proceedings of the 14th International IEEE Conference on Intelligent Transportation Systems (ITSC).

[3] Tiaprasert K., Zhang Y., Wang X.B., Zeng X. (2015). Queue length estimation using connected vehicle technology for adaptive signal control. IEEE Trans. Intell. Transp. Syst..

[4] Huang M., Xiong N.N., Vasilakos A.V., Liu A., Wang T. (2018). A low-latency communication scheme for mobile wireless sensor control systems. IEEE Trans. Syst. Man Cybern. Syst..

[5] Hao P., Ban X. (2015). Long queue estimation for signalized intersections using mobile data. Transp. Res. Part B Methodol..

[6] Liu X., Liu Y., Xiong N.N., Liu A., Shen H., Huang C. (2018). Construction of large-scale low-cost delivery infrastructure using vehicular networks. IEEE Access.

[7] Alkheir A.A., Aloqaily M., Mouftah H.T. (2018). Connected and autonomous electric vehicles (CAEVs). IT Prof..

[8] Li L., Wen D., Yao D. (2014). A survey of traffic control with vehicular communications. IEEE Trans. Intell. Transp. Syst..

[9] Xu H., Ding J., Zhang Y. Queue length estimation at isolated intersections based on intelligent vehicle infrastructure cooperation systems. Proceedings of the 2017 IEEE Intelligent Vehicles Symposium(IV).

[10] Guo Q., Li L., Ban X.J. (2019). Urban traffic signal control with connected and automated vehicles: A survey. Transp. Res. Part C Emerg. Technol..

[11] Masini B., Bazzi A., Zanella A. (2018). A survey on the roadmap to mandate on board connectivity and enable V2V-based vehicular sensor Networks. Sensors.

[12] Li T., Xiong N.N., Gao J. (2018). Reliable code disseminations through opportunistic communication in vehicular wireless networks. IEEE Access.

[13] Dai P., Liu K., Zhuge Q., Sha E.H.-M., Lee V.C.S., Son S.H. (2016). Quality-of-experience-oriented autonomous intersection control in vehicular networks. IEEE Trans. Intell. Transp. Syst..

[14] Aloqaily M., Al Ridhawi I., Kantraci B., Mouftah H.T. Vehicle as a resource for continuous service availability in smart cities. Proceedings of the IEEE 28th Annual International Symposium on Personal, Indoor, and Mobile Radio Communications (PIMRC).

[15] Silva C., Silva L., Santos L., Sarubbi J.F.M., Pitsillides A. (2019). Broadening understanding on managing the communication infrastructure in vehicular networks: Customizing the coverage using the delta network. Future Internet.

[16] Aloqaily M., Otoum S., Al Ridhawi I., Jararweh Y. (2019). An intrusion detection system for connected vehicles in smart cities. Ad Hoc Netw..

[17] Otoum S., Kantarci B., Mouftah H.T. (2019). On the feasibility of deep learning in sensor network intrusion detection. IEEE Netw. Lett..

[18] Otoum S., Kantarci B., Mouftah H.T. (2017). Detection of known and unknown intrusive sensor behavior in critical applications. IEEE Sens. Lett..

[19] Xiaojian G., Quan Z. A traffic flow forecasting model based on BP neural network. Proceedings of the 2009 2nd International Conference on Power Electronics and Intelligent Transportation System (PEITS).

[20] Aloqaily M., Kantarci B., Mouftah H.T. A generalized framework for quality of experience (QoE)-based provisioning in a vehicular cloud. Proceedings of the 2015 IEEE International Conference on Ubiquitous Wireless Broadband (ICUWB).

[21] Iqbal R., Butt T.A., Shafique M.O., Talib M.W.A., Umer T. (2018). Context-aware data-driven intelligent framework for fog infrastructures in Internet of vehicles. IEEE Access.

[22] Aloqaily M., Kantarci B., Mouftah H.T. (2016). Multiagent/multiobjective interaction game system for service provisioning in vehicular cloud. IEEE Access.

[23] Zheng J., Liu H.X. (2017). Estimating traffic volumes for signalized intersections using connected vehicle data. Transp. Res. Part C Emerg. Technol..

[24] Wang X., Chen C., Min Y. (2018). Efficient metropolitan traffic prediction based on graph recurrent neural network. arXiv.

[25] Wu M., Xiong N.N., Tan L. (2018). An intelligent adaptive algorithm for environment parameter estimation in smart cities. IEEE Access.

[26] EI-Sayed H., Sankar S., Daraghmi Y.A., Tiwari P., Rattagan E., Mohanty M., Puthal D., Prasad M. (2018). Accurate traffic flow prediction in heterogeneous vehicular networks in an intelligent transport system using a supervised non-parametric classifier. Sensors.

[27] Goudarzi S., Kama M., Anisi M., Soleymani S.A., Doctor F. (2018). Self-organizing traffic flow prediction with an optimized deep belief network for internet of vehicles. Sensors.

[28] Jiang B., Fei Y. (2017). Vehicle speed prediction by two-level data driven models in vehicular networks. IEEE Trans. Intell. Transp. Syst..

[29] Yu D., Liu C., Wu Y., Liao S., Anwar T., Li W., Zhou C. (2019). Forecasting short-term traffic speed based on multiple attributes of adjacent roads. Knowl. Based Syst..

[30] Khan S.M., Dey K.C., Chowdhury M. (2017). Real-time traffic state estimation with connected vehicles. IEEE Trans. Intell. Transp. Syst..

[31] Darwish T., Brkar K.A. (2015). Traffic density estimation in vehicular ad hoc networks: A review. Ad Hoc Netw..

[32] Cárdenas-Benítez N., Aquino-Santos R., Magaña-Espinoza P., Aguilar-Velazco J., Edwards-Block A., Cass A.M. (2016). Traffic congestion detection system through connected vehicles and big data. Sensors.

[33] Comert G. (2016). Queue length estimation from probe vehicles at isolated intersections: Estimators for primary parameters. Eur. J. Oper. Res..

[34] Comert G. (2013). Simple analytical models for estimating the queue lengths from probe vehicles at traffic signals. Transp. Res. Part B Methodol..

[35] Comert G., Cetin M. (2009). Queue length estimation from probe vehicle location and the impacts of sample size. Eur. J. Oper. Res..

[36] Comert G., Cetin M. (2011). Analytical evaluation of the error in queue length estimation at traffic signals from probe vehicle data. IEEE Trans. Intell. Transp. Syst..

[37] Ping Y.I., Zongzhong T., Qiang Z. (2008). Consistency of input-output model and shockwave analysis in queue and delay estimations. J. Transp. Syst. Eng. Inf. Technol..

[38] An C., Wu Y.J., Xia J. (2018). Real-time queue length estimation using event-based advance detector data. J. Intell. Transp. Syst..

[39] Ban X.J., Hao P., Sun Z. (2011). Real time queue length estimation for signalized intersections using travel times from mobile sensors. Transp. Res. Part C Emerg. Technol..

[40] Cheng Y., Qin X., Jin J., Ran B. (2012). An exploratory shockwave approach to estimating queue length using probe trajectories. J. Intell. Transp. Syst..

[41] Cao J., Hu D., Wang X., Md., Qiu T.Z. Comparison of queue estimation accuracy by shockwave-based and input-output-based models. Proceedings of the 17th International IEEE Conference on Intelligent Transportation Systems (ITSC).

[42] Feng Y., Head K.L., Khoshmagham S., Zamanipour M. (2015). A real-time adaptive signal control in a connected vehicle environment. Transp. Res. Part C Emerg. Technol..

[43] Mirheli A., Tajalli M., Hajibabai L. (2019). A consensus-based distributed trajectory control in a signal-free intersection. Transp. Res. Part C Emerg. Technol..

[44] Li X., Ghiasi A., Xu Z., Qu X. (2018). A piecewise trajectory optimization model for connected automated vehicles: Exact optimization algorithm and queue propagation analysis. Transp. Res. Part B Methodol..

[45] Yang K., Menendez M. (2018). Queue estimation in a connected vehicle environment: A convex approach. IEEE Trans. Intell. Transp. Syst..

[46] Cai Q., Wang Z., Zheng L. (2014). A shockwave approach to estimating queue length at signalized intersections by fusing data of point and mobile sensors. Transp. Res. Record J. Transp. Res. Board.

[47] Badillo B.E., Rakha H., Rioux T.W., Abrams M. Queue length estimation using conventional vehicle detector and probe vehicle data. Proceedings of the 15th International IEEE Conference on Intelligent Transportation Systems.

[48] Lee S., Wong S.C., Li Y.C. (2015). Real-time estimation of lane-based queue lengths at isolated signalized junctions. Transp. Res. Part C Emerg. Technol..

[49] Yang D., Chen Y., Xin L., Zhang Y. (2013). Real-time detecting and tracking of traffic shockwaves based on weighted consensus information fusion in distributed video network. IET Intell. Transport Syst..

[50] Guler S.I., Menendez M., Meier L. (2014). Using connected vehicle technology to improve the efficiency of intersections. Transp. Res. Part C Emerg. Technol..

[51] Argote-Cabañero J., Christofa E., Skabardonis A. (2015). Connected vehicle penetration rate for estimation of arterial measures of effectiveness. Transp. Res. Part C Emerg. Technol..

[52] Banani S., Gordon S. Selecting basic safety messages to verify in VANETs using zone priority. Proceedings of the 20th Asia-Pacific Conference on Communication (APCC2014).

[53] Ma X., Zhang J., Yin X., Trivedi K.S. (2012). Design and analysis of a robust broadcast scheme for VANET safety-related services. IEEE Trans. Veh. Technol..

